# Report on the Sanitary Condition of Chicago, and the Prevalence of Diseases, from August 1st to Dec. 1st, 1865

**Published:** 1866-01

**Authors:** N. S. Davis

**Affiliations:** Prof. Practical Medicine, Chicago Medical College


ARTICLE II.

### REPORT ON THE SANITARY CONDITION OF CHICAGO, AND THE PREVALENCE OF DISEASES, FROM AUGUST 1st TO DEC. 1st, 1865.

By N. S. DAVIS, M.D., Prof. Practical Medicine, Chicago Medical College.

Read to the Chicago Medical Society, December 22d, 1865.

The last report in relation to the prevalence of disease in this city, made by the undersigned committee, ended with the 1st of August. The present is simply a continuation, from the 1st of August to the 1st of December. From the 1st to the 26th of August, the atmospheric or meteorological conditions were similar to those of the last half of July, viz., cold and wet, with a prevalence of winds from the north, north-west, and north-east. During the first eleven days of the month, the rain storms were very severe, amounting in several localities in Wisconsin, Northern and Middle Illinois, and at South Bend, Indiana, to tornadoes of sufficient force to destroy crops and unroof houses. From the 1st to the 27th of the month, there were not three successive days of ordinarily hot summer weather, with winds from the south or south-west. During the same period of time, there occurred but few attacks of diarrhœa or cholera-morbus; but dysentery and typhoid fever continued quite prevalent, the latter, more especially, during the last half of the month. From the 15th to the 19th, the weather was clear and dry, but unusually cold for the season of the year, and was accompanied by a large number of cases of catarrhal irritation, with severe cough and soreness in the chest. Many of the cases of continued fever were severe, and presented more of the characteristics of typhus than of enteric typhoid fever. During the last four days of the month, the atmosphere became hot and oppressive, with winds from the south and south-west. The same characteristics were predominant during the first half of September. This was accompanied by a marked increase in the attacks of serous diarrhœa, both in children and adults.

Some of these attacks of bowel-affection, like those occurring during the first week in July, were severe, and presented symptoms much resembling epidemic cholera, such as rice water discharges, sunken eyes, huskiness of voice, small and weak pulse, with muscular cramps. But the large majority of cases were mild. From the middle of September to the 1st of December, the weather was unusually dry, pleasant, and cool.

Continued fevers, that began to be quite prevalent during the latter part of August, increased in frequency and severity during September and October; and after the two weeks of hot Summer weather, ending with the second week in September, true malarious fevers, both of the intermittent and remittent types, prevailed to a moderate extent, but decidedly more than for several years previously.

The unusual influence of malaria, as a morbific agent, was seen in the exacerbating character of many of the cases of typhoid and typhus. During the two months last named, it was very common to meet with cases of fever that had been ushered in with a chill, and which would be characterized, during the first four or five days, by such distinct exacerbations and remissions as to induce the medical attendant to regard them as true cases of periodical fever, and, consequently, to administer quinine in efficient anti-periodic doses. Instead of arresting the progress of the disease, however, it only lessened the distinctness of the exacerbations, while the fever continued, and, during the second week, developed fully the characteristic phenomena of typhoid or typhus. The unusual indications of the influence of malaria in producing periodical fevers, and modifying the symptoms of the early stage of many cases of the continued type, has not been limited to this city, but has been general over the North-west. At least, such has been the representation of correspondents from various localities in the interior.

After the middle of September, the prevalence of bowel-affections rapidly diminished, while another disease of a severe character began decidedly to increase, especially in the south-western portion of the city. We allude to a modified form of diphtheria and diphtheritic croup. It attacked children much more frequently than adults, though the latter were not wholly exempt. In a large majority of cases, the disease commenced obscurely, and with apparent mildness. The child would appear fretful and peevish, with slight feverishness for four or five days, during which the urinary secretion was generally scanty, and the bowels moderately loose, though sometimes the reverse. When loose, the discharges were generally semi-fluid and very offensive to the smell, coupled with a loss of appetite. Soon, there would commence an unusual flow of saliva, with slight swelling of one or both cheeks, and also of the salivary glands. On examining the mouth and fauces, usually the inside of the swollen cheek, the tonsils, and, sometimes, the edges of the tongue would be seen dark-red, swollen, and the mucous membrane more or less ulcerated, but very rarely more than the slightest traces of diphtheritic exudation or false membrane. The disease, thus developed, would often continue to increase, until the cheeks, sub-maxillary, and parotic regions were much swollen, and the inflamed spots in the mouth and fauces covered with ragged and extensive ulcerations, causing the saliva and breath to be fœtid, and the deglutition to be difficult. In the meantime, the patient manifests much languor and debility, with a soft and weak pulse, and a distinct increase of febrile action during the evening and first half of the night. In a few instances, during the second week, a slight exanthematous rash was noticed on the skin, and, at a later period, purple spots on the lower extremities, and an œdematous swelling of the tops of the feet. After two or three weeks, a majority of the cases began slowly to convalesce, and ultimately recovered. In a considerable proportion of the cases, however, after the first week, the local inflammation invaded the larynx, inducing all the well-characterized symptoms of diphtheritic croup, and causing a fatal result in from one to three days. In a smaller number of cases, the disease attacked the larynx primarily, inducing the ordinary symptoms of croup, with some tumefaction in the parotid and sub-maxillary regions, and death by suffocation in from 12 to 72 hours. These cases differed in their symptoms from ordinary inflammatory and pseudo-membranous laryngitis or croup, in the lower grade of febrile action, the softer and weaker pulse, and in the tumefaction of the glands in the parotid and sub-maxillary spaces.

Most of the cases of diphtheria observed during the past autumn, differed, in some important respects, from the same disease as it prevailed epidemically in this city, and many parts of the country, five or six years since. In the cases that have come under my observation this season, there has been much less white diphtheritic exudation upon the mucous membrane of the mouth and fauces, and a greater tendency to irritable, phagadænic or spreading ulceration. In a much greater number of cases, there have been observed, this season, œdematous swellings of the feet, with purpuric spots on the legs, especially when the disease ran a protracted course. One case, that occurred recently, on South Union Street, near Bunker, presented some symptoms so peculiar that a brief description may not be devoid of interest. The patient was a young man, native of Ireland, and usually enjoying good health. In the latter part of November, he had one or two teeth filled by a dentist. In about four days after, the throat and mouth were attacked with inflammation, and speedily became covered with a thick, white, tenacious, diphtheritic exudation. The swelling extended so rapidly to all the tissues in the front and lateral parts of the neck and the face below the forehead, that when I visited him, the third day, the nose, lips, cheeks, chin, sub-lingual, submaxillary, and parotid regions were swelled to such a degree, that the skin was shining and tense, the eyelids nearly closed, the jaws fixed or capable of being separated not more than a quarter of an inch, and all the tissues affected, extremely dense or unyielding to pressure. So far as the mouth and fauces could be seen through the limited opening of the jaws, the buccal mucous membrane was covered with a white, membranous exudation, with considerable tenacious mucus rattling in the fauces, and some difficulty of deglutition. But, while the interior of the mouth thus presented the well-marked appearances of diphtheria, the color of the skin over the nose, lips, and cheeks was that of a dark erysipelatous redness, with several small vesicles upon one cheek. The pulse was moderately full, but soft and frequent; the breathing noisy from mucus in the fauces, but not difficult; the mental faculties dull, and sometimes wandering; the urine scanty, and depositing a reddish sediment, and the intestinal discharges natural. In this case, we certainly had the union or intermingling of the symptoms of both diphtheria and erysipelas. During the first four days, the patient was treated with permanganate of potassa, one grain to the ounce of water, of which a tablespoonful was given every two hours, and a wash for the mouth and throat, consisting of chlorate potassa, one and a-half drachms, tinct. belladonna, three drachms, and hydrochloric acid, fifteen drops, in four ounces of water. During these four days, the local inflammation abated, the tissues of the face and neck became softer, and some of the diphtheritic coating in the mouth became detached, but his pulse became weaker, his mind more wandering, and he was much annoyed by a harassing cough, apparently from the constant accumulation of mucus in the pharynx. The permanganate of potassa was discontinued, and twenty drops of the tincture of chloride of iron given in its place, while the cough and restlessness was allayed by a teaspoonful of the following solution every four or six hours, viz.:—

℞. Muriate of Ammonia,------------------ ℥iij.

Iodide Potassa,------------------------ ℥ij.

Sulph. Morph.,------------------------ 3 grs.

Syrup Liquorice,-------------------- ℥iv.

Mix.

The swollen parts of the face and neck were also wet with the following liniment every four or five hours, viz.:—

℞. Camph. Soap Lin.,---------------------- ℥iij.

Tinct. Iodine,--------------------------- ℥j.

Mix.

Under this treatment, aided by beef-tea, chicken broth, milk porridge, &c., for nourishment, the swelling and hardness of tissues gradually subsided; the exudations and redness disappeared from the lining of the mouth and fauces; and in about two weeks from the commencement of the attack, the patient was so far recovered as to be able to return to his home, in LaSalle.

That diphtheria, of a decidedly asthenic character, has prevailed to a considerable extent during the last three or four months, chiefly in the south-west part of the city, there can be no doubt. But the great tendency on the part of the people and of some members of the profession, to confound all cases of catarrhal angina, tonsilitis, and inflammatory croup, together with true diphtheria, under the latter title, renders it impossible to make even a proximate estimate of the number of cases in any given month.

That bowel-affections, continued fevers, and diphtheria have prevailed about in the order set forth in the preceding pages of this report, is corroborated by the imperfect statistics of mortality furnished by the health-officer of the city. Thus, in August, the whole number of deaths was 464, of which 200 were represented as having resulted from bowel-affections, 21 from continued fevers, 9 from diphtheria, and 8 from croup.

In September, the whole number of deaths was 346, of which 108 were from bowel-affections, 24 from continued fevers, 11 from diphtheria, and 14 from croup. In October, the whole number of deaths was 360, of which 45 were from bowel-affections, 28 from continued fevers, 36 from diphtheria, and 40 from croup. In November, the whole number of deaths was reported to be 299, but no details are given in regard to the supposed causes of death. It will be observed that the number of deaths attributed to diphtheria and croup in October, was three times greater than in either of the preceding months; and we think the number in November was not much less.

The south-western part of the city, where a large majority of the cases of diphtheria and croup occurred during the months of October and November, is occupied almost wholly by the laboring classes, and the streets not only have no sewers, but even the roadside ditches are almost constantly filled with slop-water, garbage, and mud.

We have associated diphtheria and croup together, because personal observation has satisfied us that full half of the deaths attributed to croup were from diphtheria extending into the larynx. It is to be regretted that practitioners and writers have not maintained a more constant and definite distinction between these two diseases. Inflammatory croup, or laryngitis, is a simple local inflammation, as much so as tonsilitis, pneumonia, or pleurisy. It is never accompanied by swelling of the glands of the neck; diphtheritic exudations upon cut or abraded surfaces in other parts of the body, and fœtid breath; or followed by albuminuria, dropsy, or paralysis; all of which, however, are the common accompaniments or sequelæ of diphtheria. Hence, the latter is a general disease, involving, primarily, a morbid condition of the blood and of the vital properties of the tissues, with a constant tendency to develope a specific grade of local inflammation, most frequently in the fauces and glands of the neck, but which may be developed in almost any of the membranous structures of the body. Consequently, there is the same propriety in maintaining the distinction between laryngitis or inflammatory croup, and diphtheria accompanied by specific inflammation in the larynx, as there is between simple tonsilitis and scarlatina accompanied by anginose symptoms. In regard to the special pathology and treatment of diphtheria, there is yet much to be learned. To be told that it is a blood disease, or dependent upon a blood poison, affords us but little satisfaction or profit, so long as we are left wholly in the dark in regard to the nature of the morbid condition of the blood, or of the poison that is supposed to pervade it. That the blood is in a morbid condition, all the general symptoms, as well as the exudations upon inflamed surfaces, clearly attest. But is such morbid condition consequent on the introduction of some subtle poison from without, or on some primary change in the properties of the organized structures by which the processes of disintegration are altered and their products returned into the blood in such condition as to render the whole mass morbid? The latter supposition appears to me much more in consonance with all the facts belonging to the history, symptoms, and consequences of the disease than the former. That we have diphtheritic and true plastic, or pseudo-membranous, exudations in two widely different, if not opposite, conditions of the blood and vital properties, is evident from well-known pathological facts. Thus, the infant, in the first few days of its existence, if it fails to have a good supply of milk, and its nutritive processes fail to become established and active, will generally have its mouth, tongue, and fauces all covered with a white diphtheritic or curdy exudation. The adult, in the last stages of chronic disease, consumption for instance, when the tissues are wasted, and the function of nutrition nearly suspended, will often have a copious white exudation over the greater part of the mucous membrane of the mouth and fauces. Certainly, no one would regard the exudation in such cases as dependent on any extraneous poison imbibed into the blood, or upon any true plasticity of that fluid. On the contrary, it is clearly the result of an extremely asthenic condition of the system, with deficiency of such constituents of the blood as naturally retain the albuminous and fibrinous constituents in a soluble state. I believe that the exudations in true diphtheria result from strictly analogous morbid conditions.

That the disease is decidedly asthenic in its nature, is claimed by almost all writers on the subject, and generally assented to by the profession. That this asthenic state depends on, or consists in, deficiency of certain elements of the blood, coupled with a depressed and perverted state of vital affinity, I am fully satisfied, although I am not yet prepared to point out the exact elements at fault. The exudations in diphtheria and other asthenic conditions of the system differ in four important particulars from the true plastic or pseudo-membranous exudations resulting from simple acute inflammation. First, they appear and disappear with great rapidity, often several times during the same sickness. Second, they are accompanied by a tendency to sanious and offensive discharges from, and often ulcerations of, the membranes on which they appear. Third, the diphtheritic exudation itself, as seen under the microscope, presents cells of smaller size, arranged more in rows, and more resembling pus cells or globules, than the plastic exudations of simple inflammation. Fourth, the diphtheritic exudations all tend to ultimate dissolution, while the plastic exudations of simple inflammation tend to completeness of organization, and often become permanently identified with the original tissues.

If the foregoing statements are correct, they leave no room for doubts in regard to the propriety of maintaining a clear distinction between diphtheria complicated with specific inflammation of the larynx, and simple laryngitis or inflammatory croup. They are not only diverse in their pathology, but equally so in the indications they present for remedial treatment—the one requiring tonics, restoratives, and nutrients, the other, depletives, alteratives, and abstinence.

For nearly 30 years, we have been in the habit of meeting a greater or less number of cases of acute laryngitis or croup during the cold part of every year, and we have regarded the prompt administration of an emetic, followed by leeches to the neck, mercurial alteratives, alkaline nauseants, and such agents as speedily lessen the plasticity of the blood and the morbid sensitiveness of the solids, as affording the only reasonable hope of effecting a resolution of the inflammation, and ensuring the safety of the patient. But whoever applies the same remedies to the treatment of diphtheria, whether accompanied by exudadations in the fauces, larynx, or elsewhere, will certainly have no reason to boast of his success in restoring the health of his patients. On the contrary, in the early stage of the latter disease, we must endeavor to correct the depraved condition of the blood, increase its capacity for the absorption of oxygen, and sustain the vital affinity of the tissues by the free use of permanganate of potassa, or the chlorates of potassa and soda acidulated with hydrochloric acid, followed, in the latter stages, by the tincture of the chloride of iron, quinine, &c., with the inhalation of such vapors as tend to allay irritation of the mucous membranes, and dissolve the adherent exudations. Bland and simple nourishment must also be carefully administered throughout the whole course of the disease.

The object of this report, however, is simply to give a history of the prevalence of diseases and their connection with atmospheric and local causes, and not a discussion of their pathology or treatment. Throughout, the month of November was unusually mild and dry, and the prevalence of diphtheria and continued fevers declined both in number and severity. Hence, that month gave a gross mortality of 61 less than the preceding month. In our previous report to this Society, attention was called to the fact that we have no reliable register of the causes of death in our city. In view of expected epidemics of a certain character during the coming Spring and Summer, these statistics become of very great importance, and it is to be hoped that the Society will take immediate measures to bring about the necessary action of the city authorities.

All of which is respectfully submitted.

